# Bacterial Supplements Significantly Improve the Growth Rate of Cultured *Asparagopsis armata*

**DOI:** 10.1007/s10126-025-10440-1

**Published:** 2025-03-14

**Authors:** Jiasui Li, Lucien Alperstein, Masayuki Tatsumi, Rocky de Nys, Jadranka Nappi, Suhelen Egan

**Affiliations:** 1 Centre for Marine Science and Innovation, School of Biological, Earth and Environmental Sciences, Faculty of Science, The University of New South Wales, Kensington, Sydney, NSW 2052 Australia; 2 Sea Forest Limited, 488 Freestone Point Road, Triabunna, TAS 7190 Australia; 3 College of Science and Engineering, James Cook University, Townsville, 4810 Australia; 4 School of Life and Environmental Sciences, Faculty of Science, The University of Sydney, Camperdown, Sydney, NSW 2006 Australia; 5 Poultry Research Foundation, The University of Sydney, Camden, Sydney, NSW 2570 Australia

**Keywords:** Microbiota manipulation, Growth-promoting bacteria, Microbiome, Seaweed beneficial microorganism, Marine macroalgae, Seaweed aquaculture

## Abstract

Seaweed aquaculture is an expanding industry with innovative applications beyond the traditional uses as human foods and phycocolloids. *Asparagopsis armata*, a red seaweed, is cultivated as a feed supplement to reduce methane emission from ruminants. The manipulation of microbiota with seaweed beneficial microorganisms (SBMs) has shown promise in enhancing disease resistance and growth in seaweeds and has potential to aid the cultivation of *A. armata*. In this study, we developed a growth assay for the rapid selection of bacteria that promote the growth of *A. armata* tetrasporophytes. We tested bacterial strains from the genera *Phaeobacter* and *Pseudoalteromonas* for their impact on the growth of *A. armata*, as these bacteria have been recognized for their beneficial traits in other seaweeds. All strains significantly enhanced the specific growth rate (SGR) of *A. armata* tetrasporophytes compared to controls without bacterial treatment. Bacterial 16S rRNA gene amplicon sequencing confirmed the presence of the inoculated growth-promoting SBMs (SBM-Gs) in *A. armata* cultures with no significant impacts on the resident microbial community. Co-occurrence network analysis of the resulting communities demonstrated that the inoculated *Phaeobacter* spp. formed distinct modules, exclusively interacting with resident *Phaeobacter* species, while the *Pseudoalteromonas* sp. was absent from the network. These results demonstrate that microbial inoculation is an effective strategy for incorporating SBM-Gs into the *A. armata* microbiota to promote growth. The tested SBM-Gs may exert their influence by interacting with specific resident species or by directly affecting host physiology, resulting in minimal undesired effects on the microbiome.

**Supplementary Information:**

The online version contains supplementary material available at 10.1007/s10126-025-10440-1.

## Introduction

Seaweeds (marine macroalgae) are foundational species in coastal marine ecosystems, providing critical biodiversity hotspots and a multitude of ecosystem services (Eger et al. 2023). These include supporting food webs and fisheries (Steneck et al. 2002), nutrient removal and oxygenation (Buschmann et al. 2017), the carbon cycle (Pessarrodona et al. 2023), and offering cultural benefits such as tourism and recreation (Bennett et al. 2015). Their ecological and socio-economic importance has spurred a rapid expansion in seaweed aquaculture (Chung et al. 2017), growing at 5.8% annually from 2000 to 2022 and becoming one of the fastest-growing food sectors globally (FAO 2024). This growth is integral to achieving the United Nations’ Sustainable Development Goals (United Nations 2015), particularly in securing sustainable food sources.

The well-being and functionality of eukaryotic organisms, from humans to seaweeds, are deeply connected to the diversity and function of their microbiota (Robinson et al. 2010; Egan et al. 2013; Ren et al. 2022; Hassani et al. 2018; Hollants et al. 2013). Seaweed microbiota deliver essential nutrients and signalling molecules necessary for growth and normal development of their hosts (Singh and Reddy 2014; Wichard 2023; Ren et al. 2022; Li et al. 2023; Egan et al. 2013). For example, axenic cultures of certain green seaweeds, including *Ulva* spp., fail to develop typical morphology without key epiphytic bacteria, which excrete essential chemical compounds that stimulate seaweed cell division, differentiation, and cell wall formation (Alsufyani et al. 2020; Marshall et al. 2006; Spoerner et al. 2012). In addition, a synthetic microbial community comprising four epiphytic bacterial strains isolated from *Ulva fasciata* improved the host biomass and nutrient contents of soluble sugar and protein, with these effects associated with the upregulation of host genes involved in growth and photosynthesis (Wang et al. 2024). Similar growth effects of epiphytic bacteria are also likely for red seaweeds, for example, the presence of specific urease-producing bacteria associated with *Gracilariopsis lemaneiformis* significantly enhanced the uptake of nitrogen (Pei et al. 2024), a limiting macronutrient in many coastal habitats. Beyond growth and development promotion, manipulating microbiota with specific symbionts can prevent diseases caused by opportunistic pathogens (Li et al. 2022, 2021; Saha and Weinberger 2019). Bacteria from the *Phaeobacter* and *Pseudoalteromonas* genera, known for their antibiotic production and colonization abilities (Sonnenschein et al. 2021; Holmström and Kjelleberg 1999; Skovhus et al. 2007; Gram et al. 2015), have been found to protect against bleaching disease in red seaweeds (Li et al. 2022, 2021; Saha and Weinberger 2019) and benefit other aquatic organisms by suppressing disease (Rosado et al. 2019; Makridis et al. 2021; Offret et al. 2019; Sonnenschein et al. 2021; Pintado et al. 2023), enhancing stress tolerance (Sorieul et al. 2018) and growth (Seyedsayamdost et al. 2011; Jeon et al. 2019; Han et al. 2020; Makridis et al. 2021). Therefore, these bacteria may also provide growth benefits to seaweeds.

*Asparagopsis armata* is a seaweed that has gained substantial economic interest predominantly as a result of its ability to produce halogenated natural products, specifically haloforms, with anti-methanogenic activity (Machado et al. 2014, 2016). Feeding *Asparagopsis* spp. to ruminants has proven effects in reducing methane emissions at low dietary inclusions (reviewed by Glasson et al. 2022; Wanapat et al. 2024). Thus, enhancing the supply chain of *Asparagopsis* spp. is crucial to meet the growing bioeconomy demand (Glasson et al. 2022). *A. armata* tetrasporophytes can be successfully cultivated via land-based aquaculture (Félix et al. 2021) and therefore also represent an excellent model to investigate growth benefits of bacterial inoculants. Leveraging current knowledge of seaweed beneficial microorganisms (SBMs) that promote growth (Li et al. 2023; Singh and Reddy 2014; Ren et al. 2022), we aim to test the following: (i) whether known SBMs can act as growth-promoting SBMs, i.e., SBM-Gs, in *A. armata* tetrasporophytes, (ii) if these bacterial inoculants can be detected post inoculation, and (iii) what impact they have on the resident microbial community of *A. armata* cultures.

## Materials and Methods

### Seaweed Growth Assays

To assess the growth-promoting effects of seaweed beneficial microorganisms (SBMs) on *Asparagopsis armata* tetrasporophytes, we devised a seaweed growth assay in conjunction with 16S rRNA gene amplicon sequencing analysis (detailed methods are provided in the Supplementary Information). Briefly, *A. armata* tetrasporophytes, approximately 5 mm in diameter, were manually fragmented to an upper surface area of approximately 0.1–0.5 mm^2^ (average approximately 0.25 mm^2^) per fragment. These fragments were incubated in sterile Petri dishes with 25 mL of quarter-strength Guillard’s F/2 medium, supplemented with GeO_2_ (final concentration, 5 mg·L^−1^) to inhibit diatom proliferation (Lewin 1966). This adapted medium is denoted as F/8 medium. To avoid overcrowded fragments in later stages of the experiment, the plates were examined under a stereoscope after an overnight acclimatization, and plates with apparently unhealthy (e.g., fading), improperly sized, or overcrowded (total area per plate > 60 mm^2^) fragments were excluded. The remaining plates were randomly assigned to experimental treatments including bacterial cells suspended in F/8 medium to a final density of 10^7^ cfu·mL^−1^, or to a sterile F/8 medium-only as the control (CTR), with six biological replicates (plates) per treatments (*n* = 6). We examined four bacterial SBMs previously identified as protective for the red seaweeds *Delisea pulchra* (Li et al. 2022) and *Agarophyton vermiculophyllum* (Li et al. 2021), comprising strains *Phaeobacter piscinae* BS23 and BS52, *Phaeobacter inhibens* BS34, and *Pseudoalteromonas arabiensis* PB2-1. Growth metrics were recorded prior to the initial bacterial/CTR application (Day 1) and 5 days post the fourth weekly application (Day 26), utilizing a stereoscope (LEICA M165 FC) equipped with a 10 × eyepiece (LEICA 10450023) and a 0.63 × objective lens (PLANAPO), accompanied by a digital color camera system (Leica DFC310 FX) and LAS software v3.7.0. for photography. For each treatment or CTR, five to seven plates were included as biological replicates, and experiments were repeated three times (see Table S1 for detailed information on the experimental replication). For each biological replicate, 30 random fields were photographed as technical replicates. Image J2 software v2.9.0/1.53t (Schindelin et al. 2012) was employed to analyze the images and quantify the upper surface areas of the algal fragments, enabling specific growth rate (SGR) calculations as per the method described by Mata et al. (2017).

### Microbiota Analysis and Statistics

After growth assessment, samples (from experiment 2 and 3) underwent total DNA extraction and amplicon sequencing of the 16S rRNA gene V3-V4 region, following the methods outlined by Syukur et al. (2024) and detailed in the Supplementary Information. Sequencing was performed on an Illumina MiSeq platform following the MiSeq System User Guide (Kozich et al. 2013). The sequence data have been submitted to the BioProject database under accession number PRJNA1125579.

The resulting data were processed using a USEARCH-based pipeline as described by Li et al. (2024b) and detailed in the Supplementary Information. Briefly, the raw data were initially trimmed, quality filtered with TRIMMOMATIC version 0.38 (Bolger et al. 2014), and merged, filtered, dereplicated, chimera-removed, and clustered into amplicon sequence variants (ASVs) using USEARCH v11.0.667 and its UNOISE3 algorithm (Edgar 2010). With the UCHIME2 algorithm in USEARCH, the remaining chimeric sequences were detected and removed through reference-based comparison against the SILVA v138 (Yilmaz et al. 2014) and GTDB r214 databases (Parks et al. 2022). The resulting high-quality non-chimeric sequences were taxonomically annotated using a BLCA tool (Gao et al. 2017) against the GTDB r214 database. Finally, a feature table (ASV table) with taxonomic annotation was generated with USEARCH. To normalize uneven sequencing depths across samples, the total reads of ASVs in each sample were rarefied to the lowest number observed across all samples for subsequent analyses. Community alpha diversity indices, including the Shannon index (logarithm to base e) for diversity, observed number of ASVs/phylotypes for richness, and 1-Berger_Parker for evenness, were calculated using USEARCH.

The full-length 16S rRNA gene sequences of the inoculated bacteria from Li et al. (2022) were aligned against the ASV sequences using BioSAK v1.69.4 (https://github.com/songweizhi/BioSAK) and BLAST 2.13.0 + (McGinnis and Madden 2004). ASVs that matched with 100% identity and coverage across the V3–V4 region of the 16S rRNA gene were considered as strains closely related or belonging to the taxa of inoculated strains. To infer inter-species interactions or niche-sharing relationships (Codello et al. 2023), a bacterial co-occurrence network was established from significant correlations between pairwise relative abundances of ASVs (Spearman’s *ρ* > 0.7 or <  − 0.7, with *p*_adjusted_ < 0.05), applying a false discovery rate (FDR) control method for *p*-value correction (Benjamini and Hochberg 1995).

Hypothesis testing determined the “Treatment” effect (five levels: BS23, BS34, BS52, PB2-1, and CTR) on SGR and other microbiota parameters, including ASV relative abundance, community diversity indices, and network property indices. “Experiment” served as a random variable representing replicate experiments. Depending on the data distribution and the number of response variables, we utilized statistical models such as linear mixed-effects model (LMM), linear model (LM), generalized linear model (GLM), and multivariate GLM (mGLM), using the R packages lme4 (Bates et al. 2015) and Mvabund (Wang et al. 2012).

All data analyses and visualizations were performed using R v4.2.3. The datasets and scripts required to reproduce the study’s results are accessible in the Data Availability section. The scripts were refined and annotated with the aid of Copilot GPTs, integrated within Microsoft Edge software v125.0.2535.67.

## Results and Discussion

### Seaweed-Beneficial Bacteria Enhanced Growth of *Asparagopsis armata* Tetrasporophytes

Throughout our growth assays, all algal cultures remained healthy, and there was no outward sign of negative impacts on growth or pigmentation resulting from the bacterial treatments. Additionally, there was no obvious increase in epiphytes or bacterial overgrowth despite repeated applications of bacterial treatments (data not shown).

**Fig. 1:**
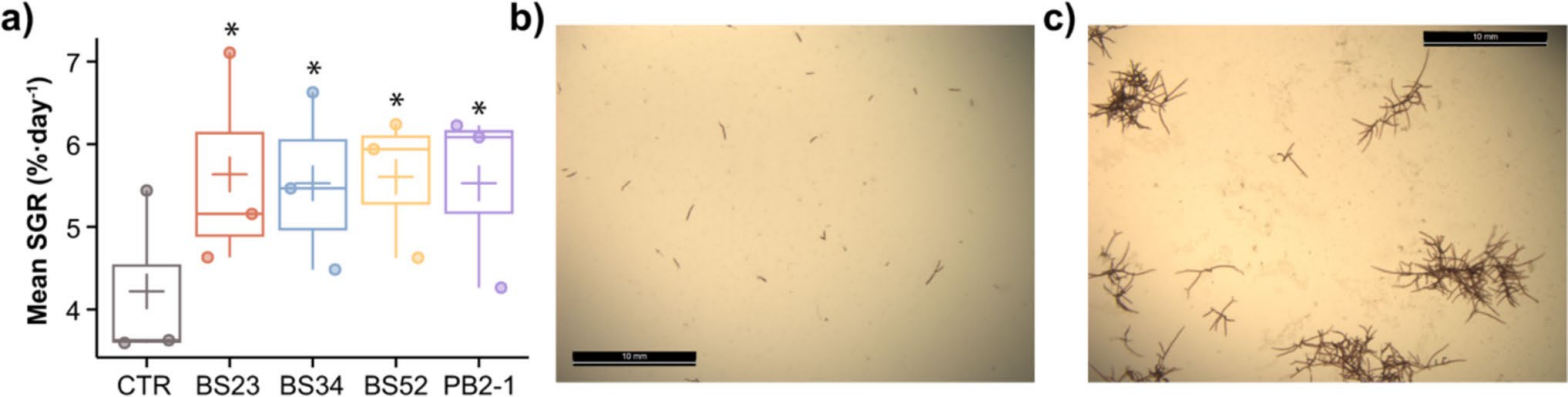
Effect of bacterial treatments on *Asparagopsis armata* growth. **a** Mean specific growth rate (SGR) of *A. armata* replicates in different treatments calculated on three independent replicate experiments. *A. armata* samples are treated by either bacterial strains *Phaeobacter piscinae* BS23 (BS23, *n* = 17), *P. piscinae* BS52 (BS52, *n* = 19), *Phaeobacter inhibens* BS34 (BS34, *n* = 17), *Pseudoalteromonas arabiensis* PB2-1 (PB2-1, *n* = 19), or sterile F/8 medium only as the control (CTR, *n* = 19). The *y*-axis shows the mean SGR in each independent replicate experiment (each dot on the graph represents one experiment, with five to seven biological replicates included for each treatment or CTR). The SGR for each biological replicate is calculated as a mean value by processing photos of 30 random fields of view, as the technical replicates. The lower and upper hinges of the boxplots correspond to the first and third quartiles, and the whiskers extend from the hinge to the 1.5 × inter-quartile range. The “ + ” on the boxplots represents the mean SGR values for each treatment or CTR from replicate experiments. The statistical difference between a bacterial treatment and CTR is denoted by a “*.” **b**, **c** Example images of algal fragments (CTR condition) viewed under a stereoscope (LEICA M165 FC) on Day 1 and Day 26, respectively. The photos are captured using LAS software v3.7.0

Previous research has demonstrated that manipulating specific bacterial epibionts can bolster the health of various red seaweed species, such as *Delisea pulchra* (Li et al. 2022) and *Agarophyton vermiculophyllum* (Li et al. 2021). Building on these insights, our current study reveals that certain bacterial strains markedly enhance the growth of *A. armata* tetrasporophytes. This enhancement is significant when contrasted with procedural controls lacking bacterial treatment (Fig. 1, Table S2). Notably, the average specific growth rate (SGR) of *A. armata* treated with *Phaeobacter piscinae* strain BS23 or *Pseudoalteromonas arabiensis* strain PB2-1 increased by 36% and 30%, respectively, relative to CTR. In one experiment, strain PB2-1 exhibited a remarkable 74% SGR improvement over the CTR (Table S2).

The practice of supplementing terrestrial plants or soils with live bacteria sourced from natural habitats can be an effective growth promoting strategy. These bacterial inoculants, commonly referred to as plant growth promoting bacteria (PGPB), work via a variety of mechanisms, including enhancing the supply of nitrogen, phosphorus, and/or iron to plants; direct synthesis or stimulating plant growth hormone production; and/or producing antimicrobials targeting plant pathogens (for more details the reader is directed to recent reviews: Li et al. 2024a; Kaminsky et al. 2019; Jaiswal et al. 2021; Negi et al. 2024; Singh et al. 2024).

Our findings suggest that this microbial inoculation approach holds promise for *A. armata* as well. *Phaeobacter inhibens* is recommended as a safe probiotic for aquaculture (Sonnenschein et al. 2021). Future research should focus on evaluating the influence of abiotic and biotic factors on the persistence and efficacy of SBM-Gs, as these have been crucial in determining the success of plant growth-promoting microorganisms in agriculture (Russ et al. 2023; Malgioglio et al. 2022).

### Composition of *Asparagopsis armata*-Associated Microbial Communities

The interactions between microbial inoculants and resident microbial communities are a critical determinant of the inoculants’ persistence and performance (Verbruggen et al. 2013; Thompson et al. 2005). The introduction of a microbial inoculant may also result in changes to resident microbiota, with yet unknown consequences (Qiu et al. 2019; Li et al. 2024a; Mawarda et al. 2020). Therefore, we conducted a 16S rRNA gene amplicon sequencing-based analysis of the bacterial community at the conclusion of the growth assays to assess (i) the abundance and prevalence of the inoculated bacteria, (ii) their impact on the native microbiota, and (iii) possible interspecies interactions between the SBM-Gs and resident bacteria.

**Fig. 2:**
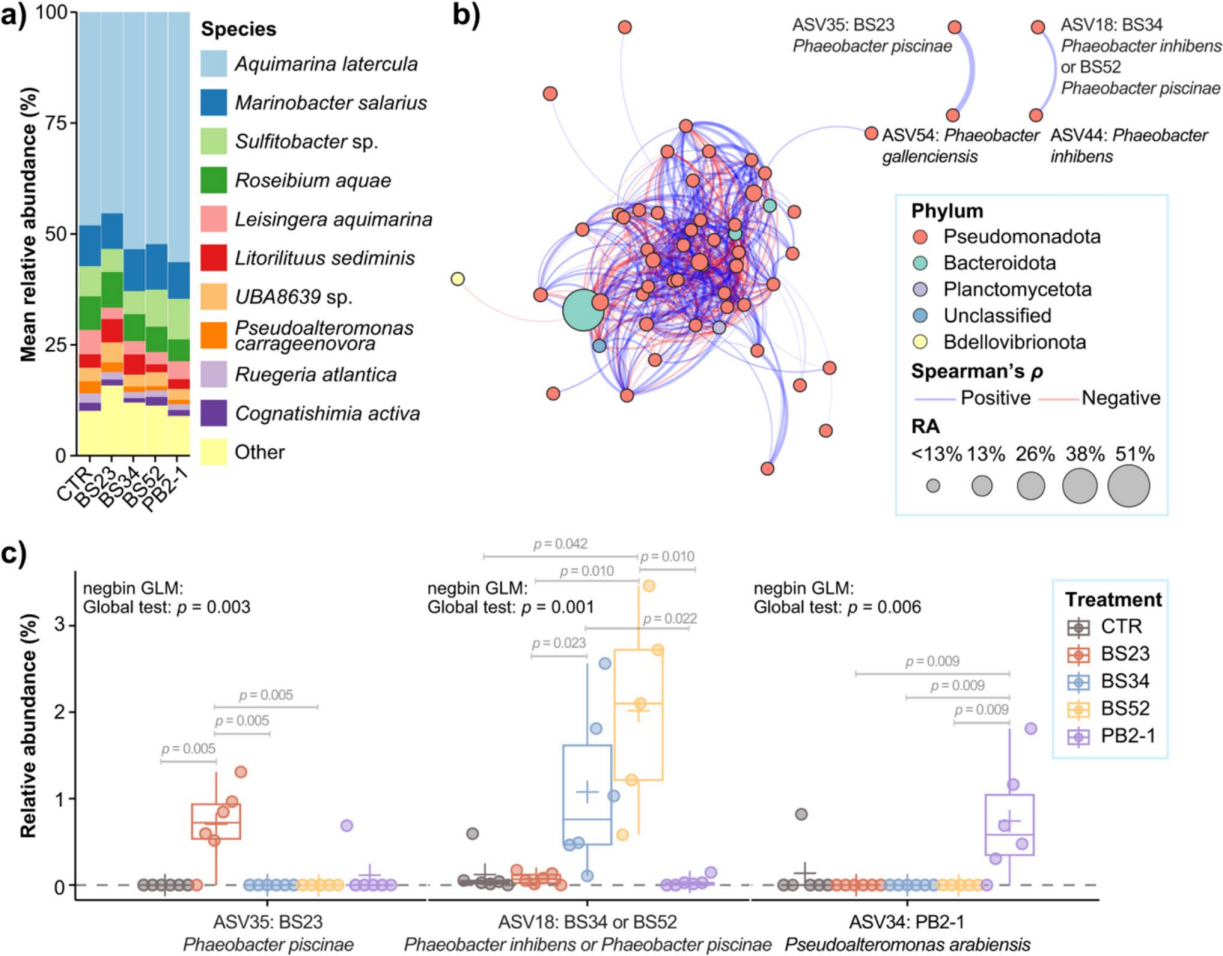
Effect of bacterial treatments on *Asparagopsis armata*-associated bacterial communities. **a** Mean relative abundances (RA) of bacterial species in *A. armata* microbiota treated with either bacterial strains *Phaeobacter piscinae* BS23 (BS23), *P. piscinae* BS52 (BS52), *Phaeobacter inhibens* BS34 (BS34), and *Pseudoalteromonas arabiensis* PB2-1 (PB2-1), or sterile F/8 medium only as the control (CTR). The mean RA is calculated based on *n* = 6 biological replicates for each treatment or CTR, except for BS52 (*n* = 5). **b** Co-occurrence network of *A. armata* microbiota constructed on Spearman’s correlations on bacterial ASV RA (*ρ* > 0.7 or <  − 0.7, and *p*_adjusted_ < 0.05). **c** RA of the inocula corresponding ASVs (16S rRNA gene V3-V4 region sequence: 100% identity and coverage) in different treatments. The lower and upper hinges of the boxplots correspond to the first and third quartiles, and the whiskers extend from the hinge to the 1.5 × inter-quartile range. The “ + ” on the boxplots represents the mean GR values calculated on *n* = 6 biological replicates for each treatment or CTR, except for BS52 (*n* = 5). negbin GLM, generalized linear model (GLM) assuming a negative binomial distribution

Our analysis produced 356,830 high-quality sequences. The high Good’s coverage (> 99.85%) and the rarefaction curves suggest that an efficient sampling depth was achieved (Table S3, Fig. S2). The epimicrobiota of cultured *A. armata* was species-sparse, with all sequences clustering into 101 ASVs, corresponding to only 81 species affiliated to eight phyla. The four most abundant species—*Aquimarina latercula* (Mean ± SD, 51% ± 17%), *Marinobacter salarius* (9% ± 12%), *Sulfitobacter* sp. (7% ± 9%; GTDB ID: *Sulfitobacter* sp001634775), and *Roseibium aquae* (6% ± 3%)—comprised over 73% of the total relative abundance (Fig. 2a). This finding contrasts with the richer bacterial species diversity found in the tetrasporophytes of *A. armata* directly collected from the environment (Parchemin et al. 2023), underscoring the influence of habitat on microbial communities.

The bacterial co-occurrence network consisted of 58 nodes (ASVs representing > 90% relative abundance) and 510 edges (Fig. 2b, Table S4), indicating a complex web of inter-taxa interactions, or niche-sharing relationships within the microbiota. Most nodes (52 nodes constituted 37% of the relative abundance) belonged to the Pseudomonadota (syn. Proteobacteria) phylum, aligning with previous studies on *Asparagopsis* spp. that found epimicrobiota were dominated by this phylum (Aires et al. 2016; Parchemin et al. 2023).

### Bacterial Supplements Significantly Enriched the Seaweed-Beneficial Bacteria in *A. armata *Tetrasporophyte Microbiota

In an ideal scenario, microbial inoculants should integrate into the host microbiota, fostering specific interactions and functions without adversely affecting the overall community structure (Mawarda et al. 2020; Xiong et al. 2013). Our research revealed that the SBM-Gs, or their taxonomically closely related species, were present within the *A. armata* tetrasporophyte-associated microbiota across their treatment groups, with presence also detected in untreated groups at lower frequencies and relative abundances (Fig. 2c). Specifically, ASV35, matching the *Phaeobacter piscinae* strain BS23, was detected in most of the samples that received the BS23 treatment (prevalence, 5/6; mean relative abundance, 0.7%) and in one sample from the PB2-1 treatment at a lower relative abundance (prevalence, 1/6; mean relative abundance, 0.1%), but was absent in all samples from CTR, BS34, and BS52 treatments. Similarly, ASV34, matching the *Pseudoalteromonas arabiensis* strain PB2-1, was present in five out of six samples treated with PB2-1 (prevalence, 5/6; mean relative abundance, 0.74%), detected in one CTR sample (prevalence, 1/6) with a lower mean relative abundance (0.14%), and absent in other treatments. Furthermore, ASV18, matching both the *Phaeobacter inhibens* strain BS34 and the *P. piscinae* strain BS52, was found in all samples receiving either treatment (in BS34: prevalence: 6/6, mean relative abundance: 1.1%; in BS52: prevalence: 5/5, mean relative abundance: 2%). ASV18 was also present in samples from the CTR (prevalence, 5/6; mean relative abundance, 0.1%), BS23 (prevalence, 5/6; mean relative abundance, 0.07%), and PB2-1 (prevalence, 4/6; mean relative abundance, 0.03%), but at significantly lower relative abundances (> tenfold less) compared to the corresponding SBM-G treatments (Fig. 2c, Table S5).

This observation of SBM-G-related ASVs present in untreated samples suggests a natural association of these SBM-Gs, or their closely related species, with *A. armata*. It is posited that microbial inoculants are more effective within their native hosts, likely due to their pre-adaptation to the ecological niches they inhabit (Jiang et al. 2023). Moreover, the relative abundances of the SBM-G-related ASVs were significantly higher in the groups that received the corresponding SBM-G treatments compared to the untreated groups (Fig. 2c, Table S5), demonstrating that microbial inoculation can effectively enrich the populations of SBM-Gs in *A. armata*-associated microbiota. The ability to proliferate in sites where the inoculants were applied is a desired trait to withstand prevailing environmental stressors and biotic competition, making them more likely to remain active in real-world conditions (Kaminsky et al. 2019; Thompson et al. 2005). The observed prevalence of our inoculants or related strains in resident microbiota of *A. armata* and the significant increase of them in populations following bacterial inoculation suggest the inoculants possess these advantageous traits as SBM-Gs.

### Bacterial Supplements Did Not Cause Community Level Shifts in *A. armata* Tetrasporophyte Microbiota

At the community level, no significant shifts were observed in response to bacterial treatments in terms of the community composition, structure, alpha diversity, and ASV co-occurrence network properties (Supplementary Fig. S3, Table S6-S8). These findings are consistent across various taxonomic levels (Table S6-S8) and align with previous research indicating that certain *Phaeobacter* spp. can colonize the green alga *Ulva ohnoi* without altering the alga-associated bacterial communities’ diversity and composition (Pintado et al. 2023).

Although knowledge on the impact of SBMs on seaweed microbiomes is limited, evidence from terrestrial plants suggests that multiple factors influence the fate of the microbial inoculants and communities, including growth and spread traits of the inoculants, niche availability, complexity and composition of the resident microbiome, and the host’s chemical responses to inoculation (Mawarda et al. 2020; Li et al. 2024a; Mallon et al. 2015a; Wen et al. 2023; Dittmann et al. 2019; Kurkjian et al. 2021). For instance, soil communities with high species richness and niche overlap with the invader tend to reduce invasion success (Wei et al. 2015), while a low level of niche overlap may predict better establishment (Mallon et al. 2015a, b; Russ et al. 2023). Consequently, the low species richness of microbiota of the land-based aquarium-cultured *A. armata* may have provided more vacant niches (e.g., unconsumed resources) that allowed the tested SBM-Gs to thrive without needing to suppress or displace resident members, thus causing negligible overall effects on the community. This hypothesis is supported by observations that strain PB2-1 (ASV34) was absent from the co-occurrence network nodes, while the *Phaeobacter* strains BS23 (ASV35), BS34, and/or BS52 (ASV18) formed distinct network modules, connecting exclusively with indigenous *Phaeobacter* strains (Fig. 2b).

In addition to the possibility of shared niches, the positive associations between the inoculated and resident *Phaeobacter* spp. suggest potential synergistic relationships, which could be leveraged to enhance the survival and functions of the beneficial bacteria (Li et al. 2019; Hang et al. 2022; Tao et al. 2020). For example, the addition of *Trichoderma* bio-organic fertilizer has been shown to enrich *Aspergillus* spp. in cucumber cultivating soils, promoting plant yield, while the co-inoculation of *Aspergillus* spp. isolates with *Trichoderma* has been found to increase the growth promotion effects through synergism (Hang et al. 2022). Similarly, positive correlations between the relative abundances of a biocontrol inoculant *Bacillus* sp. and indigenous *Pseudomonas* spp. have been observed, with co-inoculation shown to suppress *Fusarium* wilt disease in bananas (Tao et al. 2020). These findings imply that the SBM-Gs, specifically BS23, BS34 and BS52, may exert their beneficial effects through synergism with specific members of the microbiota, while PB2-1 may influence the physiological traits of the seaweed directly. Future work could aim to manipulate such synergistic interactions for enhanced beneficial effects. However, it is important to note that these predicted interspecies interactions may be specific to the aquarium culture of *A. armata* tetrasporophytes used in this study. Therefore, future work should be undertaken to assess the effectiveness of the SBM-Gs on seaweeds at different life stages and under different cultivation conditions.

## Conclusions

This study developed a growth assay to explore the potential of using seaweed-associated bacteria as growth-promoting seaweed beneficial microorganisms (SBM-Gs) in cultivated red seaweed *Asparagopsis armata* at its early life stage. The results suggested that four strains—*Phaeobacter piscinae* BS23, *P. piscinae* BS52, *Phaeobacter inhibens* BS34, and *Pseudoalteromonas arabiensis* PB2-1— could be SBM-Gs for cultured *A. armata tetrasporophytes*. A hybrid methodology, including microscopy, 16S rRNA gene amplicon sequencing, and bioinformatic tools, was used to capture the shifts in microbiota following bacterial supplementation. The results support that these SBM-Gs can be successfully delivered to algal microbiota through inoculation without causing significant shifts in community structure or co-occurrence networks. Therefore, we suggest these four strains as promising candidates for further scale-up studies, focusing on their effects under different cultivation conditions, host life-stages, and potential impacts on species sharing natural environments.

## Supplementary Information

Below is the link to the electronic supplementary material.

Supplementary file1 (DOCX 683 KB)

Supplementary file2 (XLSX 36.1 KB)

## Data Availability

The sequence data have been submitted to the BioProject database under accession number PRJNA1125579. Scripts and data necessary to reproduce all statistical analyses and visualisations in this article are available at: 10.6084/m9.figshare.25709862.v1. Due to the large size of the raw microscopy image data, which included over 7,000 photos in a ZIP file exceeding 20 GB, these files have not been deposited into a public database. However, they are available upon request via correspondence.
