# Supplementary material for: Bacterial Supplements Significantly Improve the Growth Rate of Cultured Asparagopsis armata
Source: Mar Biotechnol (NY). 2025 Mar 14;27(2):65. doi: 10.1007/s10126-025-10440-1 (PMC11909060; doi:10.1007/s10126-025-10440-1)
Supplement: Supplementary file 1 — Supplementary file1 (DOCX 683 KB) [file 10126_2025_10440_MOESM1_ESM.docx]

**Supplementary Information**

Bacterial supplements significantly improve the growth rate of cultured *Asparagopsis armata*

**Journal name:** Marine Biotechnology

Jiasui Li^1,4,5^, Lucien Alperstein^2^, Masayuki Tatsumi^2^, Rocky de Nys^2,3^, Jadranka Nappi^1^ and Suhelen Egan^1^

^1^ Centre for Marine Science and Innovation, School of Biological, Earth and Environmental Sciences, Faculty of Science, The University of New South Wales, Kensington, Sydney, NSW 2052, Australia

^2^ Sea Forest Limited, 488 Freestone Point Road, Triabunna, Tasmania 7190, Australia

^3^ College of Science and Engineering, James Cook University, Townsville, 4810, Australia

^4^ School of Life and Environmental Sciences, Faculty of Science, The University of Sydney, Camperdown, Sydney, NSW 2006, Australia

^5^ Poultry Research Foundation, The University of Sydney, Camden, Sydney, NSW 2570, Australia

**Corresponding author**

Suhelen Egan,

Centre for Marine Science and Innovation, School of Biological, Earth and Environmental Sciences, Faculty of Science, The University of New South Wales, Kensington, Sydney, NSW 2052, Australia

s.egan@unsw.edu.au

**E-mail addresses all co-authors**

Jiasui Li: jiasui.li@sydney.edu.au

Lucien Alperstein: l.alperstein@unsw.edu.au

Masayuki Tatsumi: mt@seaforest.com.au

Rocky de Nys: rdn@seaforest.com.au

Jadranka Nappi: j.nappi@unsw.edu.au

Suhelen Egan: s.egan@unsw.edu.au

**Contents:**

**Supplementary Materials and Methods:**

**Sources and culture conditions of seaweed and bacteria**

*Asparagopsis armata* tetrasporophytes were sourced from Sea Forest (Triabunna) on April 27, 2022. The algae were maintained in seawater and transported to CMSI labs at UNSW, Sydney, within 24 hours. Throughout the study, the samples were consistently maintained at 17 ± 1 °C under a 12-h light/12-h dark cycle, with a photon flux density of 5-6 µmol·m^-2^·s^-1^ during the light period.

The algae were acclimated in F/8 medium for four weeks to recover from sampling stress before being utilized in growth assays. The F/8 medium, a quarter-strength Guillard’s F/2 (Cell-Hi F2P, Varicon Aqua Solutions Ltd., Worcester) medium, was prepared using 0.22 µm filtered and autoclaved sea seawater. The salinity was adjusted to 36 practical salinity units (PSU), and GeO_2_ was added to a final concentration of 5 mg·L^-1^ to suppress diatom growth (Lewin, 1966). The medium was refreshed weekly for the duration of the experiments.

We selected four seaweed beneficial microorganisms (SBMs) previously identified on *Delisea pulchra* (Li et al. 2022) and *Agarophyton vermiculophyllum* (Li et al. 2021) as potential seaweed growth-promoting microorganisms (SBM-Gs). These strains included *Phaeobacter piscinae* BS23 and BS52, *Phaeobacter inhibens* BS34, and *Pseudoalteromonas arabiensis* PB2-1. For detailed information on the originality of these bacteria, refer to Li et al. (2021). The bacteria strains were resuscitated from 30% glycerol (v/v) stocks routinely stored at -80 °C and cultured on Marine Broth 2216 (Difco) agar plates (MA) at 25°C for routinely culture.

**Seaweed growth assay**

To assess the impact of bacterial treatments on *A. armata* growth, we established a growth assay. Initially, the algal tetrasporophytes, approximately 5 mm in diameter, were manually fragmented to yield pieces with a surface area of about 0.1-0.5 mm^2^ each. These fragments were then placed in sterile Petri dishes containing 25 mL of F/8 medium (Supplementary Fig. S1). It is crucial to note that the initial amount and size of tetrasporophyte fragments are vital for the success of the assay. A starting biomass exceeding 60 mm^2^ per plate or algal fragments larger than 0.5 mm^2^ per fragment can lead to overcrowded fragments in later days, which can hinder accurate growth measurement. Thus, after an overnight acclimatization period, the plates were examined under a stereoscope, and those with overcrowded, improperly sized, fading, or dead fragments were excluded. The remaining plates with viable fragments were randomly assigned to one of four bacterial treatments or to an F/8 medium-only as the control/CTR, with six biological replicates (plates) per treatment (*n* = 6).

A fresh colony of bacteria on MA plates was picked to be cultured in Marine Broth 2216 (MB) at 180 rpm and 25°C to reach log-phase growth. Subsequently, 500 µL of bacteria suspension was prepared in F/8 medium (OD_600_ = 0.1) and inoculated to each plate to achieve a final concentration of 10^7^ cfu·mL^-1^, following the procedures outlined by Li et al. (2022). The same volume of sterile F/8 medium was added for the CTR group. The treatment process, including medium change and inoculation for seaweed samples, was repeated weekly for four weeks (Day 1, 7, 14 and 21).

Size assessments were conducted on Day 1, post-water change and prior to any treatments, and again on Day 26 in the same manner. Each plate was viewed using a stereoscope (LEICA M165 FC) equipped with a 10× eyepiece (LEICA 10450023) and a 0.63× objective lens (PLANAPO), along with a digital colour camera system (Leica DFC310 FX) and LAS software v3.7.0. For each treatment or CTR, five to seven plates were included as biological replicates, and the whole experiments were repeated three times (see Table S1 for detailed information on the experimental replication). We captured 30 random fields per plate as technical replicates.

Image J2 software v2.9.0/1.53t (Schindelin et al. 2012) was used to quantify the number of fragments and measure the area of each. The specific growth rates (SGRs) of *A. armata* were calculated using the formula described by Mata et al. (2017):

$$SGR\left( \%\cdot{Day}^{-1} \right)=\frac{Ln\left( \frac{B_{f}}{Bi} \right)}{T}\times100$$

where *B_f_* and *B_i_* represent the final (Day 26) and initial (Day 1) surface areas, respectively, and *T* demotes the number of days in culture.

**16S rRNA gene-based amplicon sequencing and data processing**

After the final size assessment, seaweed samples were collected for DNA extraction and amplicon sequencing, to investigate the colonization by inoculated bacteria and their impact on surface-associated bacterial communities. The culture medium was removed, and algal fragments were thoroughly rinsed three times with 10 mL of sterile 1× PBS (phosphate-buffered saline) to eliminate loosely associated bacteria. The cleaned fragments were then resuspended in 3 mL of sterile 1× PBS. We centrifuged 600 μL of this suspension at 5,000× *g* for one minute, discarding the supernatant, and stored the resulting algal pellets at -80°C for subsequent DNA extractions.

DNA extraction, library preparation, and amplicon-sequencing were performed according to the methods detailed in Syukur et al. (2024). Total DNA was extracted using the DNeasy PowerSoil Kit (Qiagen, Chadstone, VIC, Australia) according to the manufacturer's instructions. The NOCHL primer pairs with Illumina overhang adaptors, NOCHL_F: 5’-tcgtcggcagcgtcagatgtgtataagagacagCCTACGGGNGGCWGCAG-3’ and NOCHL_R: 5’-gtctcgtgggctcggagatgtgtataagagacagCMGGGTATCTAATCCKG-3’, were used to amplify the V3-V4 region of the 16S rRNA gene (Thomas et al. 2020). The amplicons underwent paired-end sequencing (2 × 300 bp) on an Illumina MiSeq platform at the Ramaciotti Centre for Genomics (UNSW, Australia), following the MiSeq System User Guide (Kozich et al. 2013).

The sequencing data were processed and analysed using the code from Li et al. (2024). Initially, raw data were trimmed and quality filtered with TRIMMOMATIC version 0.38 (Bolger et al. 2014). Paired reads were merged, filtered, and dereplicated using USEARCH v11.0.667 (Edgar, 2010), and high-quality sequences were clustered into amplicon sequence variants (ASVs) (Prodan et al. 2020) using the UNOISE3 algorithm. Chimeric sequences were identified and removed both *de novo* during ASV clustering and through reference-based comparison against the SILVA v138 (Yilmaz et al. 2014) and GTDB r214 databases (Parks et al. 2022), using the UCHIME2 algorithm (Edgar, 2016). Taxonomic classification of non-chimeric sequences was performed with the BLCA tool (Gao et al. 2017) against the GTDB r214 database. Finally, the processed sequences were mapped onto ASV sequences to calculate the count distribution of each ASV across the samples.

**Microbiota diversity and composition analyses**

We visualised the sequencing depth with rarefaction curves and estimated sampling efficiency using Good’s coverage indices, using the R package vegan (Oksanen et al. 2019) and QsRutils (Zhang et al. 2017), respectively. To normalize uneven sequencing depths across samples, we subsampled the count data to the lowest number of reads observed using USEARCH. All subsequent analyses were conducted on this normalized dataset.

For community alpha diversity, we calculated indices such as Shannon index using a natural logarithm (Shannon_e) for diversity, observed ASVs/phylotypes for richness, and 1-Berger_Parker for evenness, using the -alpha_div command in USEARCH. To assess the treatment effects on the relative read abundances, prevalence of inoculated bacteria, and indigenous communities, these data were fitted into different models for hypothesis testing (details provided in the Statistical analyses section below).

**Co-occurrence network construction**

To construct a robust co-occurrence network and avoid spurious correlations, only ASVs observed in a minimum of three samples and with an average relative abundance of at least 0.001% across all samples were included. Spearman’s rank correlation coefficients (*ρ*) were computed among pairwise ASVs using the *rcorr* function in R package Hmisc (Harrell Jr & Harrell Jr, 2019). Correlations with a Spearman’s *ρ* above 0.7 or below -0.7, with an adjusted *p*-value below 0.05 following the False Discovery Rate (FDR) control method (Benjamini & Hochberg, 1995) were considered significant and used to construct the network. The network’s topological features, including average degree (representing network complexity), graph density, average clustering coefficient, average path length, and modularity, were computed using the igraph R package (Csardi & Nepusz, 2006). The networks were visualised using the Cytoscape software v3.10.2 (Shannon et al. 2003).

**Statistical analyses**

Throughout all analyses the ‘Treatment’ (five levels: BS23, BS34, BS52, PB2-1, and CTR) was designated as the fixed factor, while ‘Experiment’ (three levels: E1-E3 for growth assay, and two levels: E2-E3 for microbiota analyses) served as the random factor to account for batch variance. SGRs and alpha diversity indices were analysed using linear mixed-effect models (LMM) or, when LMMs proved overly complex, linear models (LM) specifying both treatment and experiment as fixed factors were employed. These analyses were conducted using the *lmer* and *lm* functions from the lme4 R package (Bates et al. 2015).

The relative read abundance of ASVs was analysed using either a generalised linear model (GLM) for univariate data or multivariate GLM (mGLM) for multivariate community data, assuming a negative binomial distribution with the Mvabund package (Wang et al. 2012). For presence/absence data, we assumed a binomial distribution. ASVs that significantly responded to bacterial treatments were identified through univariate GLM within the mGLM framework, with FDR-adjusted *p*-values below 0.05 (Li et al. 2022).

Multiple comparisons were performed to assess the impact of bacterial treatments on SGRs (i.e., by comparing them against the CTR) using the function *glht* in R package multcomp (Hothorn et al. 2008). *P*-values from these comparisons were adjusted using the FDR control method (Benjamini & Hochberg, 1995). Unless specified otherwise, all data analyses and visualizations were performed using R version 4.2.3.

**Supplementary Figures (Figure S1-S3)**


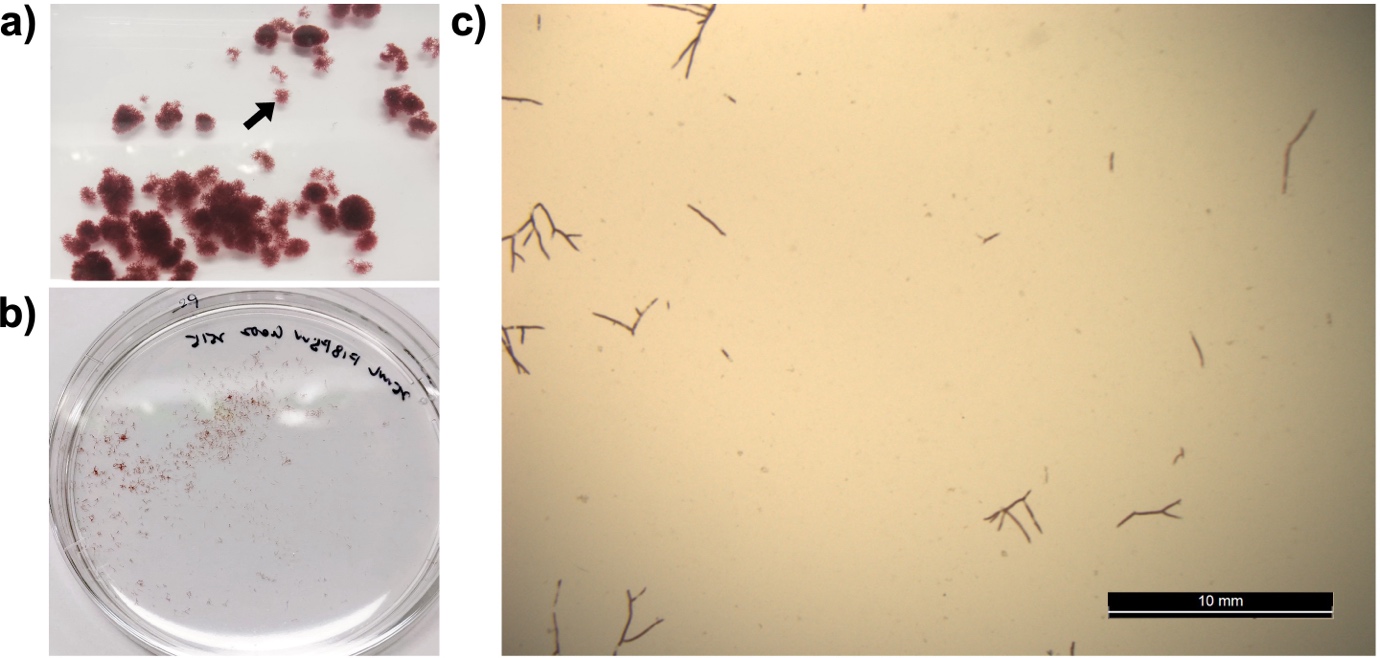


**Figure S1.** Examples of *Asparagopsis armata* fragments used in growth assays. **a),** tetrasporophytes of *A. armata*. The arrow directs to one tetrasporophyte of the ideal size (approximately 5 mm in diameter) for fragmentation. **b),** Seaweed fragments in a petri dish (bottom diameter: 84 mm) containing 25 mL of F/8 medium. **c),** Seaweed fragments viewed under a stereoscope (LEICA M165 FC). The photo is captured using LAS software v3.7.0.


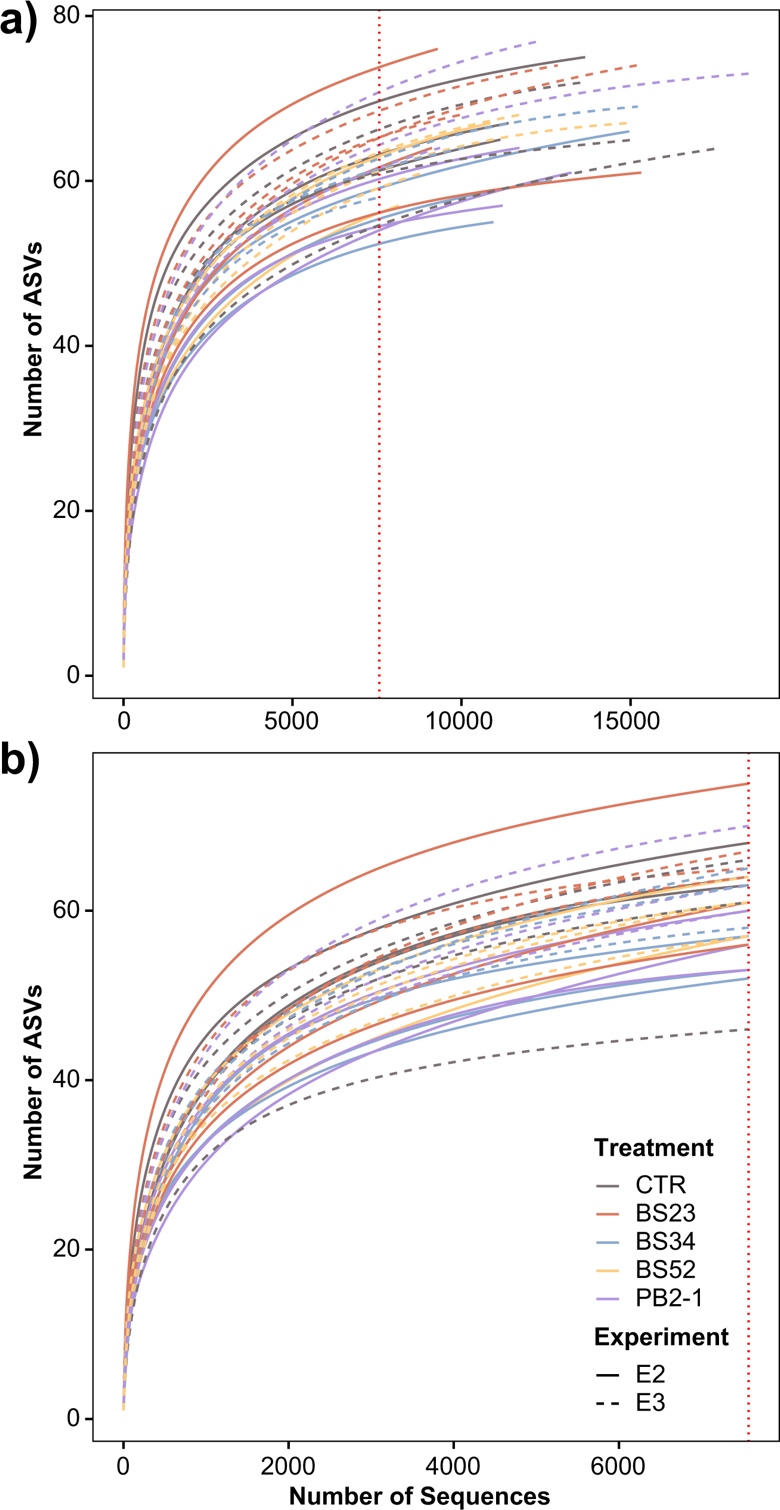


**Figure S2.** Rarefaction curves. The bacterial ASV numbers are plotted against the numbers of quality-filtered sequences before **(a)** and after **(b)** being subsampled to the lowest sequences yielded (the vertical dot lines indicate 7,570) for the bacterial communities of *Asparagopsis armata* tetrasporophytes.


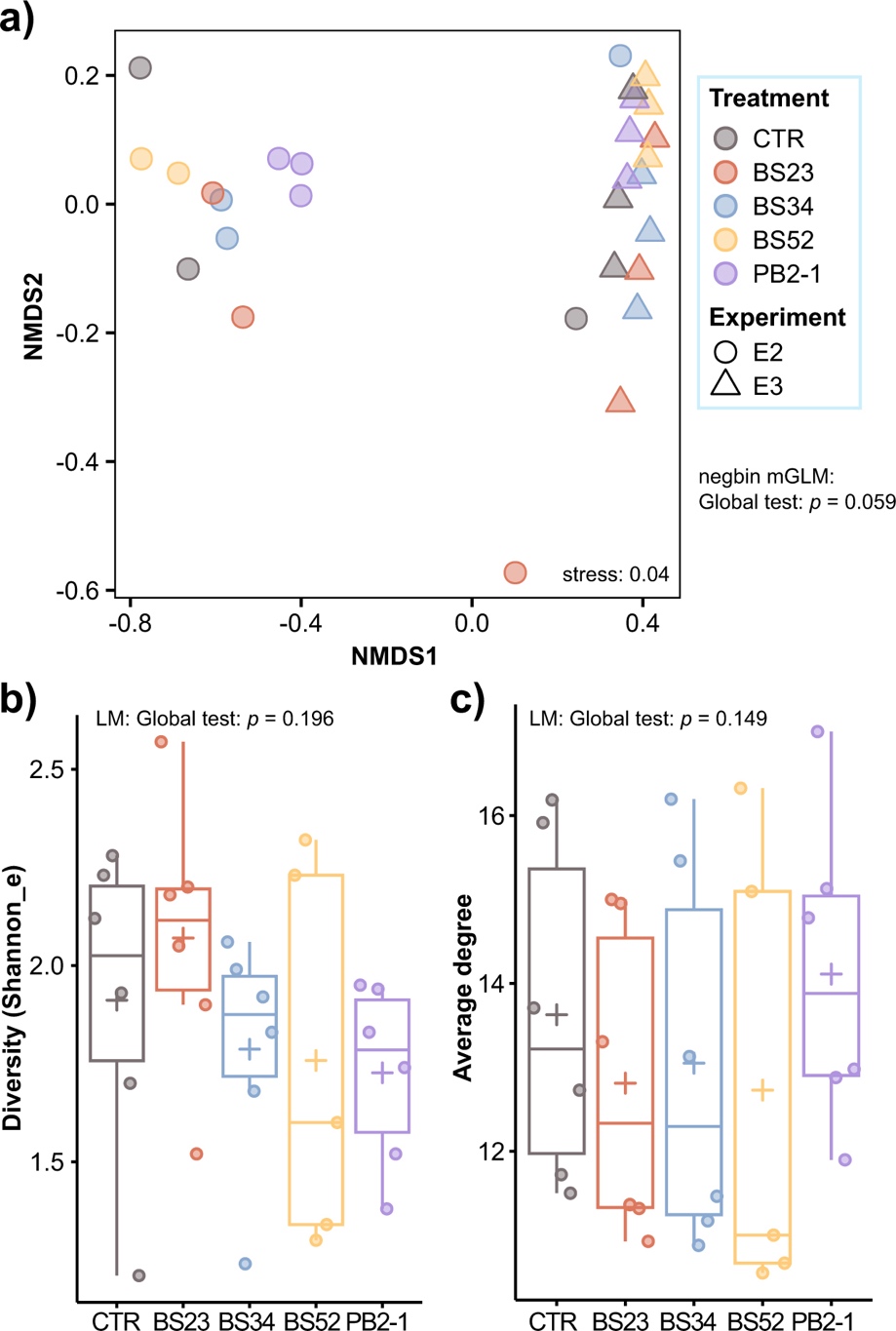


**Figure S3.** Effect of bacterial treatments on the community structure, diversity, and co-occurrence network complexity of *Asparagopsis armata*-associated microbiota. *A. armata* microbiota were treated with either bacterial strains *Phaeobacter piscinae* BS23 (BS23), *P. piscinae* BS52 (BS52), *Phaeobacter inhibens* BS34 (BS34), and *Pseudoalteromonas arabiensis* PB2-1 (PB2-1), or sterile F/8 medium only as the control (CTR). **a)**, Non-metric multidimensional scaling (nMDS) plot of the Bray-Curtis dissimilarities showing the community structure at the ASV level, **b)**, Shannon index, logged to base e, representing the community alpha diversity. **c)**, Average degree representing the network complexity of bacterial communities associated with *A. armata* samples. Statistical significance of the treatment effect was annotated on the plots, with detailed information available in Table S6-S8. Abbreviations: negbin mGLM: multivariate generalised linear model (GLM) assuming a negative binomial distribution; LM: linear model.

**Supplementary Tables (Table S1-S8)**

**Table S1.** Replication details for *Asparagopsis armata* growth assays

| **Treatment** | **Experiment ID^*^** | **Date of experiment (Day 1)** | **Number of biological replicates** |
| --- | --- | --- | --- |
| CTR | E1 | 7/10/2022 | 6 |
| BS23 | E1 | 7/10/2022 | 5 |
| BS34 | E1 | 7/10/2022 | 5 |
| BS52 | E1 | 7/10/2022 | 6 |
| PB2-1 | E1 | 7/10/2022 | 6 |
| CTR | E2 | 15/10/2022 | 6 |
| BS23 | E2 | 15/10/2022 | 6 |
| BS34 | E2 | 15/10/2022 | 6 |
| BS52 | E2 | 15/10/2022 | 6 |
| PB2-1 | E2 | 15/10/2022 | 6 |
| CTR | E3 | 21/11/2022 | 7 |
| BS23 | E3 | 21/11/2022 | 6 |
| BS34 | E3 | 21/11/2022 | 6 |
| BS52 | E3 | 21/11/2022 | 7 |
| PB2-1 | E3 | 21/11/2022 | 7 |

*E1-E3: experiment 1 to 3

**Table S2.** Multiple comparison results of mean specific growth rates fitted in either a linear mixed-effects model (LMM) where the ‘Treatment’ is specified as a fixed factor and the ‘experiment’ (three levels representing the replicate experiments E1-E3) as a random factor [formula: lmer(Growth_rate ~ Treatment + (1 | Experiment), data = Combined_data, REML = F)]; or linear modes (LM) for the separate experiments [formula: lm(Growth_rate ~ Treatment, data = Replicate_experiment_data)]. The *p*-values for multiple comparisons were adjusted using an FDR (False Discovery Rate) control method (Benjamini & Hochberg, 1995).

*Tests on a LMM for the combined data from three replicate experiments (E1-E3)*

| **Compared treatment** | **SGR improvement (%)** | ***z* value** | ***p*_unadjusted_** | ***p*_adjusted_** |
| --- | --- | --- | --- | --- |
| BS23 - CTR | 35.80 | 3.740 | 0.000184 | 0.000622 |
| BS34 - CTR | 31.98 | 3.321 | 0.000896 | 0.000896 |
| BS52 - CTR | 32.46 | 3.606 | 0.000311 | 0.000622 |
| PB2-1 - CTR | 30.31 | 3.361 | 0.000777 | 0.000896 |

*Tests on a LM for data from E1*

| **Compared treatment** | **SGR improvement (%)** | ***t* value** | ***p*_unadjusted_** | ***p*_adjusted_** |
| --- | --- | --- | --- | --- |
| BS23 - CTR | 28.97 | 1.094 | 0.28533 | 0.2853 |
| BS34 - CTR | 52.09 | 1.970 | 0.06097 | 0.0813 |
| BS52 - CTR | 64.46 | 2.591 | 0.01634 | 0.0327 |
| PB2-1 - CTR | 73.54 | 2.909 | 0.00791 | 0.0317 |

*Tests on a LM for data from E2*

| **Compared treatment** | **SGR improvement (%)** | ***t* value** | ***p*_unadjusted_** | ***p*_adjusted_** |
| --- | --- | --- | --- | --- |
| BS23 - CTR | 30.70 | 0.522 | 0.00371 | 0.0149 |
| BS34 - CTR | 21.88 | 0.522 | 0.03138 | 0.0628 |
| BS52 - CTR | 14.71 | 0.522 | 0.13644 | 0.1819 |
| PB2-1 - CTR | 11.76 | 0.522 | 0.22726 | 0.2273 |

*Tests on a LM for data from E3*

| **Compared treatment** | **SGR improvement (%)** | ***t* value** | ***p*_unadjusted_** | ***p*_adjusted_** |
| --- | --- | --- | --- | --- |
| BS23 - CTR | 42.15 | 3.740 | 0.00728 | 0.0291 |
| BS34 - CTR | 23.42 | 3.321 | 0.11643 | 0.1552 |
| BS52 - CTR | 27.55 | 3.606 | 0.05908 | 0.1182 |
| PB2-1 - CTR | 17.36 | 3.361 | 0.22037 | 0.2204 |

**Table S3. General characteristics of the 16S rRNA amplicon sequencing data of bacterial communities associated with** ***Asparagopsis armata* tetrasporophytes. Seaweeds were treated with different bacteria strains:** ***Phaeobacter piscinae* BS23 (BS23), *P. piscinae* BS52 (BS52), *Phaeobacter inhibens* BS34 (BS34), *Pseudoalteromonas arabiensis* PB2-1 (PB2-1), or sterile F/8 medium only as the control (CTR). The samples were collected five days after the final treatment (*n* = 6 for each treatment or CTR, except for BS52 where only five replicates were available).** (Extended Excel data file)

**Table S4.** Information on the 58 nodes in the bacterial co-occurrence network (Extended Excel data file)

**Table S5.** Analysis of variance (ANOVA) assessing the effect of bacterial treatment on the relative abundance (RA) of ASVs corresponding to the inoculated bacteria within the ***Asparagopsis armata*-associated bacterial community on Day 26 post-inoculation. The RAs of ASV35 (representing *Phaeobacter piscinae* BS23), ASV18 (*Phaeobacter inhibens* BS34 and/or *P. piscinae* BS52), and ASV34 (*Pseudoalteromonas arabiensis* PB2-1) were fitted into** generalised linear models (mGLM) assuming a negative binomial distribution, where each ASV was treated as a separate GLM. The ‘Experiment’ and ‘Treatment’ were fitted sequentially and as interactive factors. Further pairwise comparisons between treatments and the control (CTR) were performed when a significant effect was observed for the treatment factor. *P*-values were calculated using 1,000 iterations via PIT-trap resampling. For multiple comparisons, *p*-values are adjusted using a free stepdown method.

*Global tests*

| **Factor** | ***Df*** | **ASV35** | | **ASV18** | | **ASV34** | |
| --- | --- | --- | --- | --- | --- | --- | --- |
|  |  | ***Deviance*** | ***p*** | ***Deviance*** | ***p*** | ***Deviance*** | ***p*** |
| Experiment | 1, 27 | 0.072 | 0.555 | 3.688 | 0.060 | 0.101 | 0.534 |
| Treatment | 4, 23 | 16.688 | 0.003 | 28.305 | 0.001 | 16.130 | 0.006 |
| Experiment × Treatment | 4, 19 | 6.147 | 0.080 | 17.516 | 0.031 | 6.297 | 0.060 |

*Pairwise comparisons*

| **Compared treatment** | **ASV35** |  | **ASV18** |  | **ASV34** |  |
| --- | --- | --- | --- | --- | --- | --- |
|  | ***Deviance*** | ***p*** | ***Deviance*** | ***p*** | ***Deviance*** | ***p*** |
| BS23 - CTR | 15.456 | 0.005 | 0.377 | 0.781 | 1.536 | 0.664 |
| BS34 - CTR | 0.000 | 1.000 | 6.607 | 0.126 | 1.536 | 0.664 |
| BS52 - CTR | 0.000 | 1.000 | 9.440 | 0.042 | 1.349 | 0.664 |
| PB2-1 - CTR | 1.535 | 0.716 | 1.765 | 0.781 | 1.303 | 0.664 |
| BS23 - BS34 | 15.456 | 0.005 | 12.127 | 0.023 | 0.000 | 1.000 |
| BS23 - BS52 | 14.093 | 0.005 | 17.266 | 0.010 | 0.000 | 1.000 |
| BS23 - PB2-1 | 1.570 | 0.425 | 1.099 | 0.781 | 14.601 | 0.009 |
| BS34 - BS52 | 0.000 | 1.000 | 1.764 | 0.781 | 0.000 | 1.000 |
| BS34 - PB2-1 | 1.535 | 0.716 | 13.068 | 0.022 | 14.601 | 0.009 |
| BS52 - PB2-1 | 1.348 | 0.716 | 16.601 | 0.010 | 13.264 | 0.009 |

**Table S6.** ANOVA tests analysing the effect of bacterial treatment on the community structure at ASV and different taxonomic levels (i.e., from species to phylum). The relative read abundances of ASVs or other phylotypes were fitted into multivariate generalised linear models (mGLM) assuming a negative binomial distribution, where each ASV or phylotype was treated as a separate GLM. The ‘Experiment’ and ‘Treatment’ were fitted sequentially and as interactive factors. Further one-factor analyses of the ‘Treatment’ factor were performed within each experiment when a significant interaction was observed between the factors (i.e., *p* < 0.05 for ‘Experiment × Treatment’). *P*-values were calculated using 1,000 iterations via PIT-trap resampling. For multiple comparisons, *p*-values are adjusted using a free stepdown method. (Extended Excel data file)

**Table S7.** ANOVA tests assessing the effect of bacterial treatment on the community diversity (represented by Shannon_e indices), richness (Observed phylotypes), and evenness (1-Berger-Parker) of ***Asparagopsis armata***-associated epimicrobiota at different phylogenetic levels (i.e., from ASV to phylum). The alpha diversity indices were fitted into linear models (LM) where the ‘Experiment’ and ‘Treatment’ were fitted sequentially and as interactive factors. The *lm* and *anova* functions in the stats R package were used for model fitting and *p*-value calculation, respectively. (Extended Excel data file)

**Table S8.** ANOVA tests assessing the effect of bacterial treatment on the co-occurrence network characteristics. The data were fitted into linear models (LM) where the ‘Experiment’ and ‘Treatment’ were fitted sequentially and as interactive factors. The *lm* and *anova* functions in the stats R package were used for model fitting and *p*-value calculation, respectively. (Extended Excel data file)

**References:**

Bates D, Mächler M, Bolker B, Walker S (2015) Fitting linear mixed-effects models using lme4. J Stat Softw 67:1-48. https://doi.org/10.18637/jss.v067.i01

Benjamini Y, Hochberg Y (1995) Controlling the false discovery rate: a practical and powerful approach to multiple testing. J R Stat Soc Series B Stat Methodol 57:289-300. https://doi.org/10.1111/j.2517-6161.1995.tb02031.x

Bolger AM, Lohse M, Usadel B (2014) Trimmomatic: a flexible trimmer for Illumina sequence data. Bioinformatics 30:2114-2120. https://doi.org/10.1093/bioinformatics/btu170

Csardi G, Nepusz T (2006) The igraph software. Complex Syst 1695:1-9. https://doi.org/10.5281/zenodo.7682609

Edgar R (2016) UCHIME2: improved chimera prediction for amplicon sequencing. bioRxiv. https://doi.org/10.1101/074252

Edgar RC (2010) Search and clustering orders of magnitude faster than BLAST. Bioinformatics 26:2460-2461. https://doi.org/10.1093/bioinformatics/btq461

Gao X, Lin H, Revanna K, Dong Q (2017) A Bayesian taxonomic classification method for 16S rRNA gene sequences with improved species-level accuracy. BMC Bioinformatics. https://doi.org/10.1186/s12859-017-1670-4

Harrell Jr FE, Harrell Jr MFE (2019) Package ‘hmisc’. CRAN2018 2019:235-236. https://doi.org/10.32614/CRAN.package.Hmisc

Hothorn T, Bretz F, Westfall P (2008) Simultaneous inference in general parametric models. Biometr J Math Methods Biosci 50:346-363. https://doi.org/10.1002/bimj.200810425

Kozich JJ, Westcott SL, Baxter NT, Highlander SK, Schloss PD (2013) Development of a dual-index sequencing strategy and curation pipeline for analyzing amplicon sequence data on the MiSeq Illumina sequencing platform. Appl Environ Microbiol 79:5112-5120. https://doi.org/10.1128/AEM.01043-13

Lewin J (1966) Silicon metabolism in diatoms. V. Germanium dioxide, a specific inhibitor of diatom growth. Phycologia 6:1-12. https://doi.org/10.2216/i0031-8884-6-1-1.1

Li J, Weinberger F, Saha M, Majzoub ME, Egan S (2021) Cross-host protection of marine bacteria against macroalgal disease. Microb Ecol 84:1288-1293. https://doi.org/10.1007/s00248-021-01909-2

Li J, Majzoub ME, Marzinelli EM, Dai Z, Thomas T, Egan S (2022) Bacterial controlled mitigation of dysbiosis in a seaweed disease. ISME J 16:378-387. https://doi.org/10.1038/s41396-021-01070-1

Li J, Saha M, Majzoub ME, Yang T, Chu H, Thomas T, Weinberger F, Egan S (2024) Non-selective microbiota reduction after the elicitation of a seaweed’s immune response. Environ Microbiol Rep. https://doi.org/10.1111/1758-2229.13268

Mata L, Lawton RJ, Magnusson M, Andreakis N, de Nys R, Paul NA (2017) Within-species and temperature-related variation in the growth and natural products of the red alga *Asparagopsis taxiformis*. J Appl Phycol 29:1437-1447. https://doi.org/10.1007/s10811-016-1017-y

Oksanen J, Blanchet FG, Friendly M, Kindt R, Legendre P, McGlinn D, Minchin PR, O’Hara R, Simpson GL, Solymos P (2019) Package ‘vegan’. Community Ecol Package, version 2. https://github.com/vegandevs/vegan

Parks DH, Chuvochina M, Rinke C, Mussig AJ, Chaumeil P-A, Hugenholtz P (2022) GTDB: an ongoing census of bacterial and archaeal diversity through a phylogenetically consistent, rank normalized and complete genome-based taxonomy. Nucleic Acids Res 50:D785-D794. https://doi.org/10.1093/nar/gkab776

Prodan A, Tremaroli V, Brolin H, Zwinderman AH, Nieuwdorp M, Levin E (2020) Comparing bioinformatic pipelines for microbial 16S rRNA amplicon sequencing. PLOS ONE. https://doi.org/10.1371/journal.pone.0227434

Schindelin J, Arganda-Carreras I, Frise E, et al. (2012) Fiji: an open-source platform for biological-image analysis. Nat Methods 9:676-682. https://doi.org/10.1038/nmeth.2019

Shannon P, Markiel A, Ozier O, Baliga NS, Wang JT, Ramage D, Amin N, Schwikowski B, Ideker T (2003) Cytoscape: a software environment for integrated models of biomolecular interaction networks. Genome Res 13:2498-2504. https://doi.org/10.1101/gr.1239303

Syukur S, Richmond J, Majzoub ME, Nappi J, Egan S, Thomas T (2024) Not all parents are the same: diverse strategies of symbiont transmission in seaweeds. Environ Microbiol. https://doi.org/10.1111/1462-2920.16564

Thomas F, Dittami SM, Brunet M, Le Duff N, Tanguy G, Leblanc C, Gobet A (2020) Evaluation of a new primer combination to minimize plastid contamination in 16S rDNA metabarcoding analyses of alga‐associated bacterial communities. Environ Microbiol Rep 12:30-37. https://doi.org/10.1111/1758-2229.12806

Wang Y, Naumann U, Wright ST, Warton DI (2012) mvabund– an R package for model-based analysis of multivariate abundance data. Methods Ecol Evol 3:471-474. https://doi.org/10.1111/j.2041-210X.2012.00190.x

Yilmaz P, Parfrey LW, Yarza P, Gerken J, Pruesse E, Quast C, Schweer T, Peplies J, Ludwig W, Glöckner FO (2014) The SILVA and “All-species Living Tree Project (LTP)” taxonomic frameworks. Nucleic Acids Res 42:D643-D648. https://doi.org/10.1093/nar/gkt1209

Zhang B, Penton CR, Xue C, Quensen JF, Roley SS, Guo J, Garoutte A, Zheng T, Tiedje JM (2017) Soil depth and crop determinants of bacterial communities under ten biofuel cropping systems. Soil Biol Biochem 112:140-152. https://doi.org/10.1016/j.soilbio.2017.04.019
